# Thermal Drift Correction for Laboratory Nano Computed Tomography via Outlier Elimination and Feature Point Adjustment

**DOI:** 10.3390/s21248493

**Published:** 2021-12-20

**Authors:** Mengnan Liu, Yu Han, Xiaoqi Xi, Siyu Tan, Jian Chen, Lei Li, Bin Yan

**Affiliations:** Henan Key Laboratory of Imaging and Intelligent Processing, PLA Strategic Support Force Information Engineering University, Zhengzhou 450001, China; lmn1242@163.com (M.L.); hy007hy007@126.com (Y.H.); xqxi2021@163.com (X.X.); tan.siyu@hotmail.com (S.T.); kronhugo@163.com (J.C.); leehotline@163.com (L.L.)

**Keywords:** nanoscale computed tomography (nano-CT), thermal drift correction, outlier elimination

## Abstract

Thermal drift of nano-computed tomography (CT) adversely affects the accurate reconstruction of objects. However, feature-based reference scan correction methods are sometimes unstable for images with similar texture and low contrast. In this study, based on the geometric position of features and the structural similarity (SSIM) of projections, a rough-to-refined rigid alignment method is proposed to align the projection. Using the proposed method, the thermal drift artifacts in reconstructed slices are reduced. Firstly, the initial features are obtained by speeded up robust features (SURF). Then, the outliers are roughly eliminated by the geometric position of global features. The features are refined by the SSIM between the main and reference projections. Subsequently, the SSIM between the neighborhood images of features are used to relocate the features. Finally, the new features are used to align the projections. The two-dimensional (2D) transmission imaging experiments reveal that the proposed method provides more accurate and robust results than the random sample consensus (RANSAC) and locality preserving matching (LPM) methods. For three-dimensional (3D) imaging correction, the proposed method is compared with the commonly used enhanced correlation coefficient (ECC) method and single-step discrete Fourier transform (DFT) algorithm. The results reveal that proposed method can retain the details more faithfully.

## 1. Introduction

Computed tomography (CT), which is a nondestructive technique to obtain structural information inside objects, is widely used in cultural relic detection, life sciences, and other industrial applications [1]. However, small changes in the relative position of source-turntable-detector can seriously affect the image quality of CT [2,3]. In particular, the projection misalignment caused by thermal drift can deteriorate the reconstruction quality and reduce the achievable spatial resolution of nano-CT [4,5,6]. The slices reconstructed by the misaligned projections contain serious blur and double-edge artifacts [7]. Therefore, the correction of thermal drift artifacts of great significance for boosting the practical applications of nano-CT [8,9].

The projection alignment method based on short reference scan was proposed by Sasov [10] in 2008. The method consists of three steps. (a) The second scan is carried out with a larger rotation step to obtain the reference projection. (b) The relative position relationship is calculated based on the features of the main and reference projections. (c) The alignment of projection is achieved. Feature extraction is the most challenging step because the features are directly related to the accuracy of drift calculation.

At present, there are three common methods to align the main projection with respect to the reference projection: intensity based method, frequency domain based method, and feature based method. The intensity based method constructs the similarity measure through the gray scale of projection and calculates the extreme value of the similarity measure by using the optimization algorithm to get the translation parameters. Evangelidis et al. [11] proposed an image registration method called enhanced correlation coefficient (ECC) method based on entropy correlation coefficient as the objective function and established a new iterative scheme for nonlinear optimization problems with simple closed form solutions to improve the calculation speed. The frequency domain based method aligns the projections with the relationship between the spatial movement and the parameters in the frequency domain. Manuel et al. [12] proposed a fast alignment method based on the matrix-multiply discrete Fourier transform (DFT). This method is called the single-step DFT algorithm. Its essence is that the matrix multiplication of 2D DFT to initialize the peak position of cross correlation. Consequently, the computational time is greatly reduced without sacrificing accuracy.

The feature based method uses the similarity constraint of position and direction of the feature points extracted by local descriptors to align the projections, such as scale invariant feature transform (SIFT) [13], speeded up robust features (SURF) [14], and binary robust independent elementary features (BRIEF) [15]. However, the initial features may contain a large number of mismatched points in practical applications due to the ambiguities in similarity constraint, which affects the realization of high-precision projection alignment, so it is necessary to eliminate the mismatched points [16]. Two-stage constraint-based strategy is a popular method to solve the mismatching problem [17,18,19]. In the first stage, initial matching features of image pairs are obtained by using local descriptors. In the second stage, geometric constraints of feature points are used to eliminate the outliers. The remaining features are used to construct matching parameters between image pairs. Locality preserving matching (LPM) [20] and random sample consensus (RANSAC) [21] are the commonly used methods at present. In the artifact correction of nano-CT, the projection alignment method based on feature points also requires the two-stage strategy. Since the drift process only involves horizontal and vertical movement, a rigid model is considered [22,23]. Although these methods have achieved satisfactory results in many cases, the projection of nano-CT contains significant noise and varying luminance, so the traditional two-stage strategy still needs to be optimized.

In this study, a method based on outlier elimination and position adjustment is proposed to align the main projection by the reference projection for reducing or even eliminating the thermal drift artifact of nano-CT. The proposed method can effectively eliminate outliers with high accuracy. Further, an elimination model of outliers based on the geometric position of features and structural similarity (SSIM) [24] of projection is established, which can evaluate the feature points accurately. The local SSIM between the neighborhood images of feature points is used to adjust the position. Two-dimensional (2D) transmission experiments and three-dimensional (3D) reconstruction experiments reveal that the proposed method has remarkable accuracy, and it can preserve more details of the image as compared to the existing methods.

The rest of this paper is organized as follows. Section 2 introduces the proposed method in detail. The 2D transmission experiment and 3D reconstruction experiment are described in Section 3. In Section 4, the feasibility and accuracy of method are verified by experiments and comparison with other methods. Finally, the study is concluded in Section 5.

## 2. Method

The reference projections are acquired with a larger rotation step after obtaining the main projections. The proposed method is used to calculate the drifts between the main and the reference projections. The drift of main projection without reference projection is estimated by cubic spline interpolation of adjacent drift.

**Figure 1:**
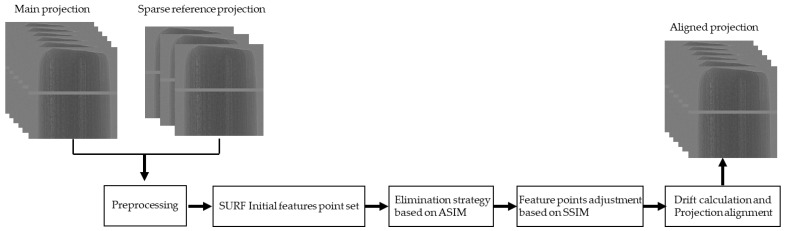
Workflow of the proposed method.

The workflow of the proposed method is shown in Figure 1, which consists of four steps: projection pretreatment, acquisition of initial features, feature point elimination based on feature angle and SSIM (ASIM), and feature point position adjustment based on SSIM. First, the main projection-reference projection image pairs are preprocessed by denoising and histogram equalization to improve the image quality and contrast. SURF is used to generate the initial features of the projections. Then, ASIM is used for feature elimination to reduce the influence of outliers on the drift calculation. Finally, the SSIM in the feature point neighborhood is used to reposition the feature.

### 2.1. Alignment Model of Main Projection and Reference Projection

The alignment model of main projection and reference projection is considered as a rigid transformation model since drift is a slowly varying translation process [25].

(1) $$\left[\begin{array}{l} x_{main}\\ y_{main} \end{array}\right]=\left[\begin{array}{l} x_{ref}\\ y_{ref} \end{array}\right]+\left[\begin{array}{l} d_{x}\\ d_{y} \end{array}\right],$$

where $\left(x_{main},y_{main}\right)$ is the coordinate of the feature point in the main projection feature set. $\left(x_{ref},y_{ref}\right)$ is the coordinate of the corresponding feature point in the reference projection feature set. $d_{x}$ and $d_{y}$ represent the horizontal and vertical rigid drifts, respectively.

### 2.2. Rough Elimination of Outliers Based on Feature Angle

SURF is a fast and robust feature description method which consists of two main parts: feature generation and matching. The purpose of feature generation is to determine feature point locations and descriptors. Matching is achieved by the Euclidean distance of the feature points.

It is assumed that the matching feature point sets have been extracted by SURF. The feature point sets of the main and reference projections are denoted by $T_{main}$ and $T_{ref}$, respectively. In the scanning process of nano-CT, the initial point set is interfered by different noise distributions and brightness [16] due to the long-time interval between the main and reference projections (usually a complete main scanning period) [10]. The outliers affect the calculation results of drift. Therefore, it is necessary to identify the inliers and outliers in the initial point set to establish a reliable matching relationship.

The proposed elimination strategy includes rough elimination and refined elimination. The purpose of rough elimination is to identify and eliminate obvious outliers for accelerating the operational efficiency of refined elimination.

**Figure 2:**
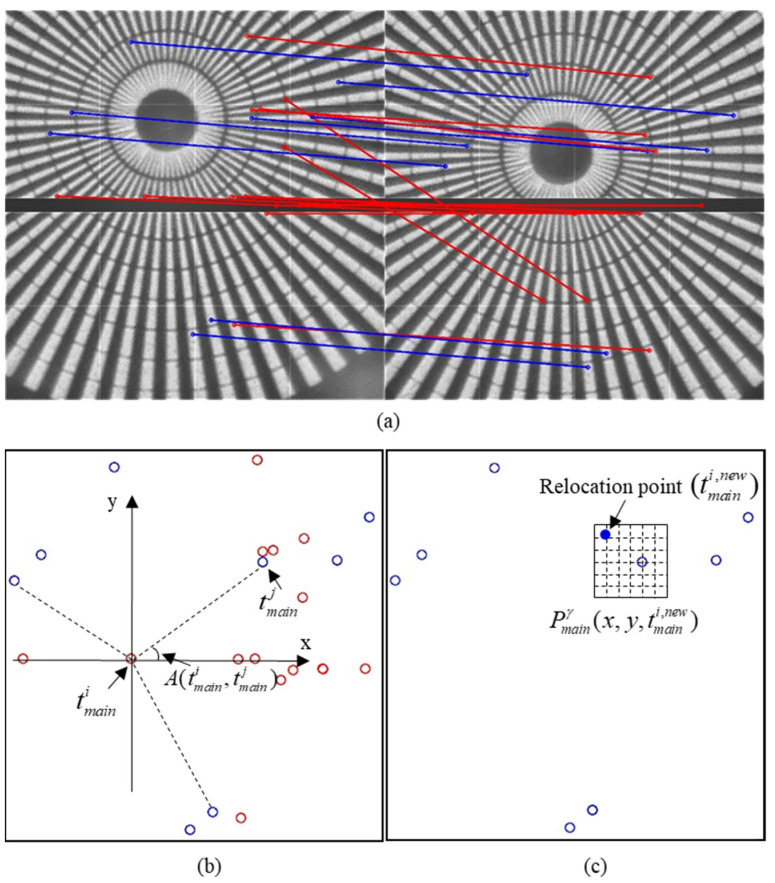
Schematic diagram of feature elimination and relocation based on ASIM. (**a**) Initial feature matching relationship, where blue and red lines represent the inliers and outliers, respectively. (**b**) Calculation of the feature angle $A(t_{main}^{i},t_{main}^{j})$ between $t_{main}^{i}$ and $t_{main}^{j}$. Only three dots are connected here for clarity. (**c**) Process of feature position adjustment, where the hollow circles represent the position of original features and the solid circles represent the new optimal location.

First, the feature points $t_{main}^{i}=(x_{main}^{i},y_{main}^{i})$, $t_{main}^{j}=(x_{main}^{j},y_{main}^{j})$ in the main feature point set $T_{main}=\{t_{main}^{i}\}(i=1,2\ldots K)$ and the corresponding points $t_{ref}^{i}=(x_{ref}^{i},y_{ref}^{i})$, $t_{ref}^{j}=(x_{ref}^{j},y_{ref}^{j})$ in the reference feature point set $T_{ref}=\{t_{ref}^{i}\}(i=1,2\ldots K)$ are considered. Here, K is the number of initial feature points. The angle between two points in the same point set is used to represent the geometric position. In the proposed method, this angle is called the feature angle. Based on the four feature points, the two feature angles are defined as

(2) $$\begin{array}{c} A(t_{main}^{i},t_{main}^{j})=\arctan\frac{y_{main}^{j}-y_{main}^{i}}{x_{main}^{j}-x_{main}^{i}}\\ A(t_{ref}^{i},t_{ref}^{j})=\arctan\frac{y_{ref}^{j}-y_{ref}^{i}}{x_{ref}^{j}-x_{ref}^{i}} \end{array},$$

where $A(t_{main}^{i},t_{main}^{j})$ and $A(t_{ref}^{i},t_{ref}^{j})$ represent the feature angle of $\left\{t_{main}^{i},t_{main}^{j}\right\}$ and $\left\{t_{ref}^{i},t_{ref}^{j}\right\}$, respectively. Please note that the feature angle contains the direction information of the two points. Here, $A(t_{main}^{i},t_{main}^{j})$ is taken as the example, and $t_{main}^{i}$ is used as the origin to establish the rectangular coordinate system. The angle between the line of $\left\{t_{main}^{i},t_{main}^{j}\right\}$ and the positive direction of the X axis is taken as the feature angle, as shown in Figure 2c.

Then, the feature angle is considered to identify the outliers. If the matching relationship of the feature points is accurate, the feature angles $A(t_{main}^{i},t_{main}^{j})$ and $A(t_{ref}^{i},t_{ref}^{j})$ should satisfy the following relationship:

(3) $$A(t_{main}^{i},t_{main}^{j})=A(t_{ref}^{i},t_{ref}^{j}).$$

However, it is difficult to satisfy Equation (3) due to the difference in brightness and noise distribution between the main projection and reference projection. The difference between the feature angle of $t_{main}^{i}$ and the feature angle of the corresponding point $t_{ref}^{i}$ is used as the evaluation criterion of outliers which can be expressed as

(4) $$F=\sum_{i\in T_{rough}}\sum_{j\in T_{rough}}\left|A(t_{main}^{i},t_{main}^{j})-A(t_{ref}^{i},t_{ref}^{j})\right|,$$

where F represents the outlier evaluation criterion. $T_{rough}$ is the set of inliers in rough elimination. Our goal is to construct the optimal inlier set $T_{rough}$ by minimizing the outlier evaluation criterion F.

To obtain the optimal solution of Equation (4), the feature angle similarity function $p_{i,j}$ is constructed to evaluate the difference between feature angles $A(t_{main}^{i},t_{main}^{j})$ and $A(t_{ref}^{i},t_{ref}^{j})$. $\varepsilon_{\min}$ and $\varepsilon_{\max}$ are the upper and lower limits of feature angle $A(t_{ref}^{i},t_{ref}^{j})$ that can be accepted relative to $A(t_{main}^{i},t_{main}^{j})$. Then, $p_{i,j}$ can be expressed as

(5) $$p_{i,j}=\left\{ \begin{array}{l} 1,\varepsilon_{\min}\leq A(t_{ref}^{i},t_{ref}^{j})\leq \varepsilon_{\max}\\ 0,else \end{array}\right..$$

It is extremely difficult to directly set the upper and lower limits of the feature angle, so the feature point offset limit χ is defined to calculate the values of $\varepsilon_{\min}$ and $\varepsilon_{\max}$. According to the direction of the feature angle, the limits of the feature angle can be expressed as

(6) $$\varepsilon_{\min}=\left\{ \begin{array}{l} A[(x_{main}^{i}-\chi ,y_{main}^{i}+\chi ),(x_{main}^{j}+\chi ,y_{main}^{j}-\chi )],A\in I\\ A[(x_{main}^{i}-\chi ,y_{main}^{i}-\chi ),(x_{main}^{j}+\chi ,y_{main}^{j}+\chi )],A\in II\\ A[(x_{main}^{i}+\chi ,y_{main}^{i}-\chi ),(x_{main}^{j}-\chi ,y_{main}^{j}+\chi )],A\in III\\ A[(x_{main}^{i}+\chi ,y_{main}^{i}+\chi ),(x_{main}^{j}-\chi ,y_{main}^{j}-\chi )],A\in IV \end{array}\right.$$

and

(7) $$\varepsilon_{\max}=\left\{ \begin{array}{l} A[(x_{main}^{i}+\chi ,y_{main}^{i}-\chi ),(x_{main}^{j}-\chi ,y_{main}^{j}+\chi )],A\in I\\ A[(x_{main}^{i}+\chi ,y_{main}^{i}+\chi ),(x_{main}^{j}-\chi ,y_{main}^{j}-\chi )],A\in II\\ A[(x_{main}^{i}-\chi ,y_{main}^{i}+\chi ),(x_{main}^{j}+\chi ,y_{main}^{j}-\chi )],A\in III\\ A[(x_{main}^{i}-\chi ,y_{main}^{i}-\chi ),(x_{main}^{j}+\chi ,y_{main}^{j}+\chi )],A\in IV \end{array}\right.,$$

where, I, II, III, and IV represent the quadrant to which the feature angle belongs. We construct the voting function based on the feature angle similarity function $p_{i,j}$

(8) $$G_{i}=\sum_{j=1}^{K}p_{i,j},$$

where, $G_{i}$ represents the voting function of feature point $t_{main}^{i}$.

Then, the rough elimination strategy of outliers can be expressed as

(9) $$T_{rough}=\left\{t_{main}^{i}|G_{i}\geq \lambda K,i=1,2\ldots N\right\}$$

where λ indicates the vote threshold. N is the number of feature points after rough elimination. $T_{rough}$ is the feature points after rough elimination. (9) provides a voting method for determining the best point set of (4). As mentioned above, the purpose of rough elimination is to eliminate obvious outliers (the feature point difference is greater than offset limit χ). Therefore, the loose vote threshold is set in Section 2.5.

### 2.3. Refined Elimination of Outliers Based on SSIM

In Section 2.2, the rough elimination of outliers based on feature angle was introduced. Obvious outliers were identified and eliminated by the feature point offset limit χ and vote threshold λ. However, feature points need to be further eliminated to improve the matching accuracy in high-precision rigid alignment. D is defined as the alignment vector, which can be expressed as follows:

(10) $$D_{i}=(d_{x}^{i},d_{y}^{i})=\left(x_{{}_{main}}^{i}-x_{ref}^{i},y_{{}_{main}}^{i}-y_{ref}^{i}\right),$$

where $D_{i}$ is the alignment vector of the point pair $\left\{t_{main}^{i},t_{ref}^{i}\right\}$ and $D_{i}\in D$. The coordinates of the feature points are expressed as $t_{main}^{i}=(x_{{}_{main}}^{i},y_{{}_{main}}^{i})$ and $t_{ref}^{i}=(x_{ref}^{i},y_{{}_{ref}}^{i})$.

The SSIM is used to further refine the features. Each pair of feature points provides a guide to align the projection through the alignment vector D. The SSIM between the reference projection and the main projection moved by the alignment vector can be expressed as

(11) $$SSIM\left[P_{main}(x-d_{x}^{i},y-d_{y}^{i}),P_{ref}(x,y)\right]=\frac{\left(2\mu_{m}\mu_{r}+C_{1}\right)\left(2\sigma_{m}\sigma_{r}+C_{2}\right)}{\left(\mu_{m}^{2}+\mu_{r}^{2}+C_{1}\right)\left(\sigma_{m}^{2}\sigma_{r}^{2}+C_{2}\right)}$$

where $P_{ref}(x,y)$ is the reference projection. $\mu_{m}$ and $\mu_{r}$ represent the mean of the main and reference projections, respectively. $P_{main}(x-d_{x}^{i},y-d_{y}^{i})$ is the main projection moved by the alignment vector $D_{i}$. $\sigma_{m}$ and $\sigma_{r}$ are the standard deviations of the main and reference projections, respectively. $C_{1}$ and $C_{2}$ are constant.

To improve the robustness of refined elimination, the SSIM threshold ε is set. Then, the refined feature points satisfy

(12) $$T_{refined}=\left\{(t_{m}(i),t_{r}(i))|SSIM\left[P_{main}(x,y),P_{ref}(x-d_{x}^{i},y-d_{y}^{i})\right]\geq \varepsilon \right\}$$

ASIM is used to eliminate the outliers in the SURF initial feature points. The complete procedure is summarized in Table 1.

### 2.4. Position Adjustment of Feature Points Based on SSIM

The location of feature points may not be calculated accurately by SURF due to the influence of brightness and gray distribution of projected image. The position adjustment method based on SSIM provides accurate position of feature points. $\left\{t_{main}^{i},t_{ref}^{i}\right\}$ is the refined feature set extracted in Section 2.3. The position adjustment of feature points method is introduced below.

In the main projection and reference projection, image blocks $P_{main}^{\gamma }(x,y,t_{main}^{i})$ and $P_{ref}^{\gamma }(x,y,t_{ref}^{i})$ with size γpixel×γpixel are cropped with $\left\{t_{main}^{i},t_{ref}^{i}\right\}$ as the center respectively. The main projection is moved within the image block with the step size of 0.1 pixel, and the SSIM of the new projection is calculated. $t_{main}^{i,new}$ represents the inlier $t_{main}^{i}$ after position adjustment. The optimal position of feature points is satisfied

(13) $$T_{refined\_relocation}=\left\{t_{main}^{i,new}|\max SSIM[P_{main}^{\gamma }(x,y,t_{main}^{i,new}),P_{ref}^{\gamma }(x,y,t_{ref}^{i})]\right\}$$

SSIM is used for feature point relocation. The relocation process is summarized in Table 2.

Figure 2 shows a schematic of feature angle and position adjustment. The proposed method uses the feature angle and SSIM for the elimination and position adjustment. Therefore, the proposed method is named ASIM.

### 2.5. Implementation Details

The features directly affect the calculation of drift. There are three parameters in the proposed algorithm: the feature point offset limit χ, the range of neighborhoods near the feature γ, and the vote threshold λ. The feature point offset limit χ controls the range of feature point deviation. The vote threshold λ represents the percentage of correct matching relation in Equation (5). Rough elimination method is used to eliminate the features with large deviations, which are measured by χ and λ. We set χ=0.5,γ=1.0, and λ=0.3.

## 3. Experiment

### 3.1. 2D Transmission Imaging Alignment Experiment

In the nano-CT scanning experiment, the time difference between the main and reference projections is long. Three 2D transmission imaging experiments are set up to evaluate the influence of brightness, noise level, and initial feature number on the alignment accuracy. To evaluate the effectiveness and robustness of the proposed method for high-precision matching between the main projection and the reference projection, the SURF, RANSAC, and LPM are used as comparison methods.

All the data used in the experiment are obtained from the nano-CT in Henan Imaging and Intelligent Information Processing Laboratory. To consider the difference in correction accuracy caused by different gray distributions and shapes, four groups of actual scan data are selected for testing. The samples and exposure time are listed in Table 3. The interval time represents the time difference between the main and reference projections. 

Firstly, the alignment effect under different lighting conditions is tested. The interval time of image pairs is shown in Table 3. The rotation angles of main projection and reference projection are the same.

Secondly, the number of initial features is considered to test the effect on accuracy. The samples in Table 3 are tested. The initial number of features are set to 75, 125, 175, and 225. 

Finally, the robustness of ASIM is tested. The sample projection pairs of the first and second rows in Table 3 are used to test the alignment results under Gaussian noise of 5%, 10%, and 15%.

### 3.2. 3D Reconstruction Experiment

To evaluate the accuracy of the proposed method in 3D reconstruction, the method is applied to the 3D imaging experiment of an electronic component and cabbage seed. The relevant scanning parameters are listed in the Table 4. The sample is rotated by 360° to obtain the projection of each rotation angle. The rotation step is the rotation angle difference between adjacent projections. The starting point of rotation for the main scan and the reference scan is the same. The proposed method is not only compared with the feature elimination methods (RANSAC and LPM), but also with the intensity based method (ECC) and frequency domain based method (the single-step DFT algorithm).

### 3.3. Evaluation Criteria

The root mean square error (*RMSE*), drift *calculation error*, *precision*, and *accuracy* are used to evaluate the effect of different methods on the elimination of feature points.

SURF is used to obtain the initial features. When the difference between the calculated result and the ground truth is less than 15%, the feature point is considered reliable, and the point is called an inlier. Otherwise, it is considered invalid and is called an outlier.

Here, *RMSE* is used to evaluate the absolute offset error. The *calculation error* is used to evaluate the relative offset error. *Accuracy* and *precision* represent the ability of elimination. The recall is not considered because it makes little difference to the drift calculation whether all the inliers are in the refined feature set. On the contrary, the precision represents the proportion of inliers in the refined feature set, which affects the calculation results. The evaluation criteria are calculated as follows:

(14) $$\begin{array}{l} RMSE=\sqrt{\frac{1}{2}\left[{\left(x-d_{x}\right)}^{2}+{\left(y-d_{y}\right)}^{2}\right]}\\ Calculation\_Error=\frac{1}{2}\left[abs\left(\frac{x-d_{x}}{x}\right)+abs\left(+\frac{y-d_{y}}{y}\right)\right]\\ Precision=\frac{TP}{TP+FP}\\ Accuracy=\frac{TP+TN}{TP+TN+FP+FN} \end{array}$$

## 4. Experimental Results and Discussion

The proposed method was compared with two effective feature elimination algorithms RANSAC and LPM. In the 3D imaging experiment, it was also compared with the single-step DFT algorithm, ECC. Finally, discussion was made according to the experimental results.

### 4.1. 2D Transmission Imaging Experiment

**Figure 3:**
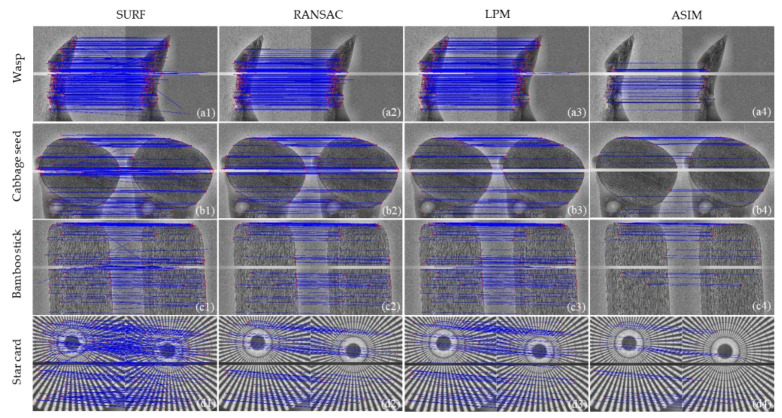
2D transmission imaging results. Rows 1 through 4 show the matching results for wasp, cabbage seed, bamboo stick, and star card, respectively. Columns 1 through 4 are the results obtained by using SURF, RANSAC, LPM, and ASIM, respectively.

Table 5 summarizes the matching results of the samples shown in Table 3 obtained using SURF, RANSAC, LPM, and the proposed method. The thermal drift eventually leads to a rigid shift of the projection. To simulate the projection drift, we move the sample. Firstly, the sample projection is acquired. Then, the X-ray source is turned off and the sample is moved so that the projection moves within the detector plane. The ground truth (in Table 5) of the projection drift is calculated by the actual distance of movement of the sample and the magnification ratio. Finally, after waiting for the interval listed in Table 3, the X-ray source is turned on and the sample projection is acquired. It is clear that *RMSE* of all three elimination methods is better than that of SURF. The *RMSE* of the three elimination methods is small in the wasp sample. Because SURF extracts a large number of initial features, the outliers are easily eliminated, which can be seen in Figure 3. In addition, the *RMSE* of the proposed method is the smallest among the three elimination methods, which indicates that the proposed method has good drift calculation accuracy and the difference between the calculated value and the true value is small.

The alignment results of the samples in Table 3 using different methods are shown in Figure 3. The feature points are marked with red circles. In Figure 3, the first, second, third, and fourth columns represent the results of the initial feature points, elimination using RANSAC, elimination using LPM, and the proposed method, respectively. The three methods can effectively eliminate the outliers. In addition, the proposed method retains less feature points than the other methods because it aims to provide accurate alignment results and ensure high accuracy, and the invalid points in the retaining features can affect the alignment result (invalid points are considered to be feature points with an error greater than 15%).

**Figure 4:**
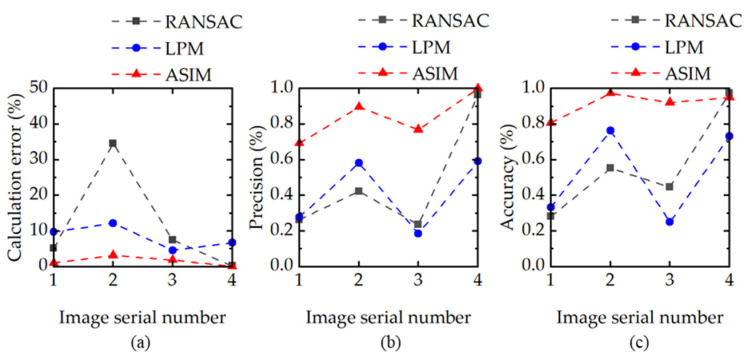
Evaluation of features eliminated by RANSAC, LPM, and ASIM. (**a**) Average error of projection alignment. (**b**) Precision of the elimination feature, which represents the correct proportion of the refined feature points. (**c**) Accuracy of the elimination feature, which represents the correct proportion of all the predicted points.

Figure 4a shows the average calculation errors of the three elimination methods. The proposed method has the smallest average errors in the four samples. Figure 4b shows the precision of the three methods, which indicates the sensitivity of the model to correct the samples. The proposed method has significant advantages because a higher percentage of feature points are correctly judged, resulting in a smaller error. Figure 4c shows the accuracy of the three methods, which is used to measure the ability to distinguish positive and negative features. It is evident from Figure 4c that the proposed method can accurately distinguish inliers from outliers, resulting in a small *RMSE* in Table 5. The accuracy and precision assess the robustness of the model, and the risk of accuracy loss is reduced when the model can correctly distinguish between inliers and outliers because more outliers increase the risk of calculation deviation.

**Figure 5:**
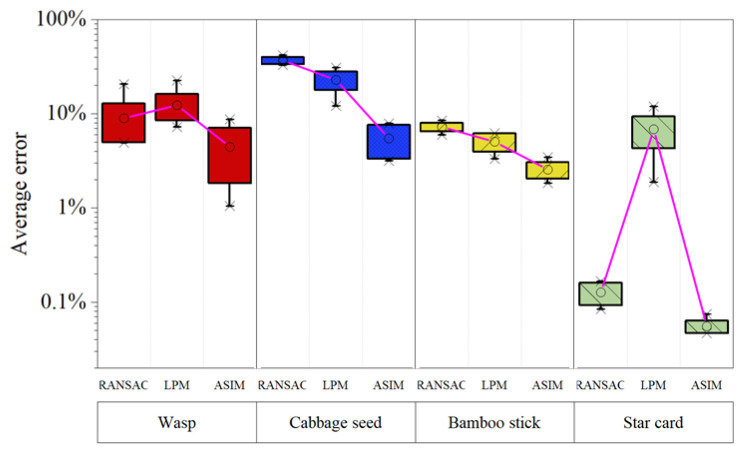
Robustness test of elimination methods. Each column corresponds to a sample listed in Table 3. In each column, the error results for different initial numbers of features are consolidated into a bin. The mean error of the calculated results is represented as ‘∘.’ The maximum and minimum value of data are represented as ‘*.’ The top and bottom borders of the box represent 0.75 and 0.25 points of data, respectively.

Different numbers of initial points are set to test the effect on the results of different elimination methods. We set four different numbers of initial points as 75, 125, 175 and 225, and the test samples are listed in Table 3. Figure 5 shows a boxplot of error results for different initial points, where each box contains errors for different points of calculation. In Figure 5, ‘∘’ represents the mean error, and the results prove that the proposed method has smaller calculation error.

**Figure 6:**
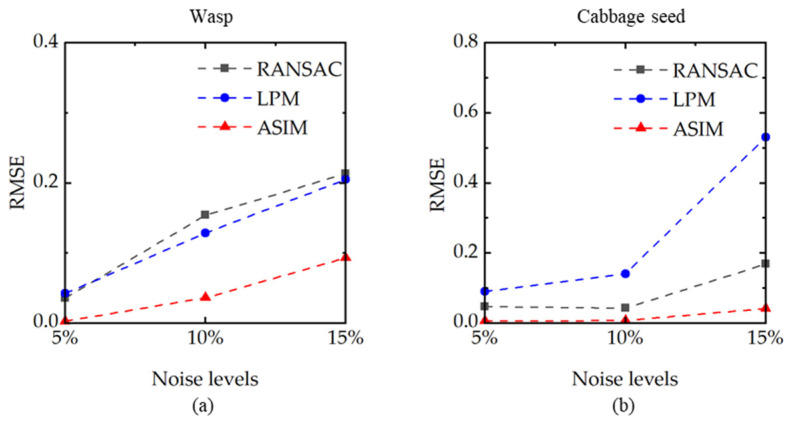
*RMSE* at different noise levels (5%, 10%, and 15%) are corrected by RANSAC, LPM, and ASIM. (**a**) The test sample is wasp. (**b**) The test sample is cabbage seed. The proposed method (red curve) achieves the most stable results.

In addition, the robustness of different methods is examined. We test the samples No.1 and No.2 in Table 3. Three different noise levels of 5%, 10%, and 15% are set, and *RMSE* is used to evaluate the error between the calculated results and the ground truth. In robustness tests, the brightness and noise distributions of image pairs are the same, so the *RMSE* shown in Figure 6 is smaller than that listed in Table 5. As the noise increases, the *RMSE* of all the three rejection methods increase, but the proposed method is still the most stable at different noise levels.

### 4.2. 3D Imaging Experiment

In this section, the correction ability of the proposed method for 3D reconstruction is experimentally evaluated. The advantages and disadvantages of the proposed method are discussed based on the experimental results.

**Figure 7:**
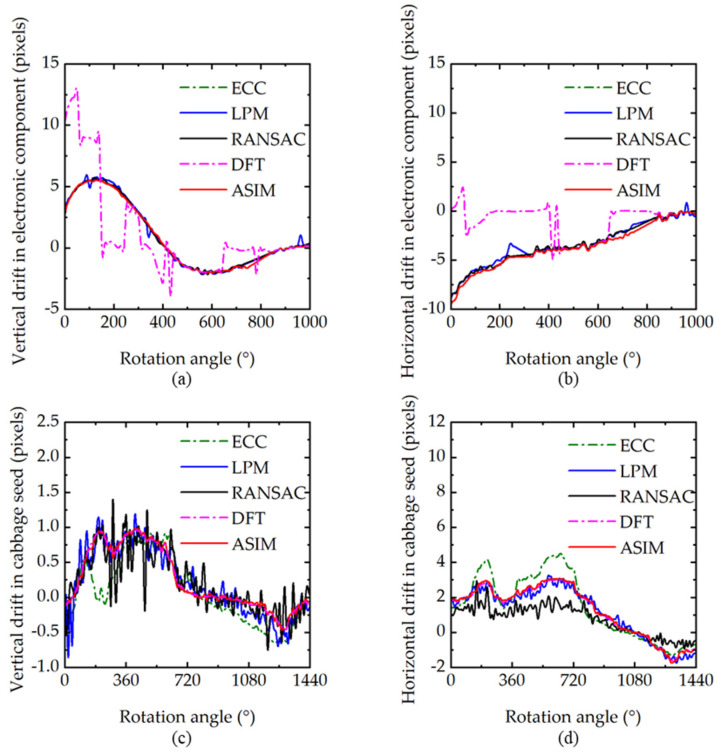
Drift calculation results. (**a**,**b**) Vertical and horizontal drifts in the electronic component scanning experiment, respectively. (**c**,**d**) Vertical and horizontal drifts in the cabbage seed scanning experiment, respectively.

Figure 7 shows the calculation results of projection drift in the 3D reconstruction correction experiment. The first line shows the calibration results of different methods in electronic component scanning. The single-step DFT algorithm (pink line in Figure 7) does not achieve good results, because the spatial resolution of electronic component imaging reaches 350 nm, and the projection noise is relatively large, which causes interference in the frequency domain registration method. The second line shows the correction results for the cabbage seed scan. The drift calculation results of LPM and RANSAC fluctuate seriously because the uncontrollable factors in the scanning process have a significant influence on the number of initial features, and RANSAC and LPM cannot eliminate the outliers completely.

**Figure 8:**
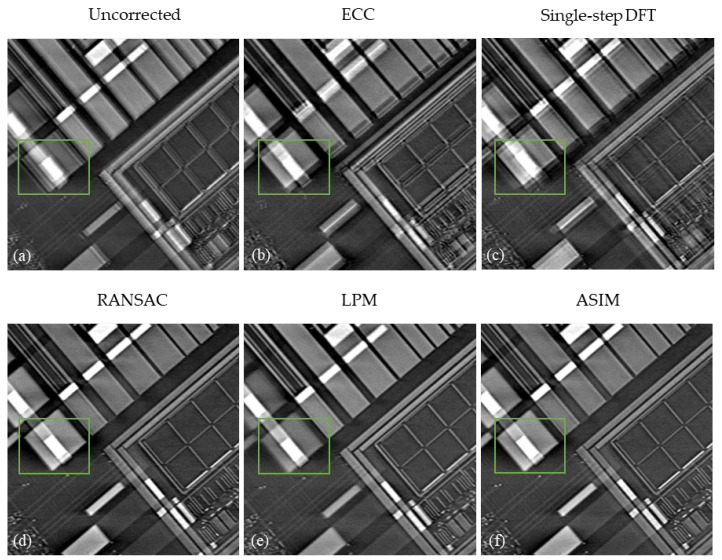
Correction results of electronic component. (**a**) Uncorrected. (**b**) ECC. (**c**) Sigle-step DFT. (**d**) RANSAC. (**e**) LPM. (**f**) ASIM.

Figure 8 shows the reconstructed slice of the electronic component corrected by different methods. Figure 8a presents the original reconstructed slices with drift artifacts. Drift artifacts have a considerable influence on the analysis of connection relationship. Serious artifacts even cause misjudgment of components. ECC provides a fast image registration method based on the entropy correlation coefficient. Figure 8b shows the reconstruction slices corrected by ECC method. Figure 8c presents the alignment results of the single-step DFT algorithm in the frequency domain. The reconstructed slices are still blurry after correction by ECC and single-step DFT algorithm. Figure 8d presents the correction results of RANSAC feature elimination method. The details of the image are well recovered. Figure 8e shows the alignment results of LPM feature elimination method. Although LPM enhances the computational speed, drift artifacts in the reconstructed slices are still obvious. Nonetheless, the reconstructed images show more information than the uncorrected ones. Figure 8f shows the corrected results of the proposed method. The fine structure on the circuit component is clearly displayed. 

**Figure 9:**
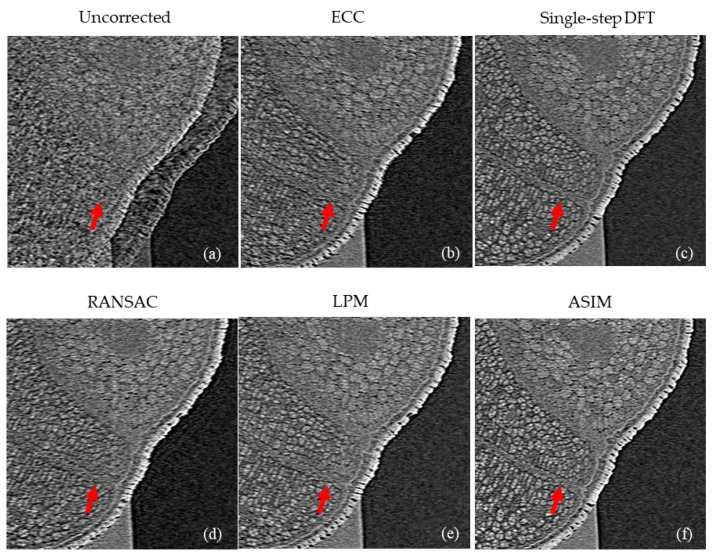
Correction results of difference alignment methods for cabbage seed. (**a**) Uncorrected. (**b**) ECC. (**c**) Sigle-step DFT. (**d**) RANSAC. (**e**) LPM. (**f**) ASIM.

Figure 9 shows the drift correction results of the cabbage seed. Drift seriously affects the 3D imaging of cabbage seed. The edge is obviously blurred, and the internal structure of cabbage seed can no longer be distinguished, as shown in Figure 9a. Figure 9b shows the correction results of ECC. The obvious drift artifact is removed, but the internal structure remains blurred. Figure 9d,e present the correction results of RANSAC and LPM, respectively. The internal structure of the seed can be clearly seen, but the position indicated by the red arrow is still unclear. The single-step DFT algorithm (Figure 9c) and the proposed method (Figure 9f) show similar correction effects. The internal structure of the seed is clearly displayed.

## 5. Conclusions

In this study, an effective reference correction method was proposed for thermal drift correction of nano-CT based on ASIM. The ASIM was used to eliminate the outliers in the initial features. The SSIM of feature point neighborhood images was used to adjust the position of feature points.

The efficacy of the proposed method in 2D transmission imaging and 3D slice reconstruction was evaluated. In 2D transmission imaging, the proposed method was compared with the commonly used RANSAC and LPM elimination methods, where samples with different gray distributions, exposure times, and shapes were used. The proposed method showed better accuracy than the other methods. In the 3D reconstruction experiment, the proposed method was compared with local feature-based ECC method and single-step DFT algorithm, and the proposed method could recover the details of the reconstructed slices well. However, the proposed method has some limitations. When the signal-to-noise ratio (SNR) of image is too low, the original feature matching fails, so the feature elimination and point adjustment can also fail. In the future, we hope to further optimize the feature matching strategy to maintain the robustness of the algorithm.

## Figures and Tables

**Table 1:** The process of outliers elimination based on ASIM.

**Input:**$\;\mathrm{The}\;\mathrm{image}\;\mathrm{pair}\;\mathrm{of}\;\mathrm{main}\;\mathrm{projection}\;P_{main}$$\;\mathrm{and}\;\mathrm{reference}\;\mathrm{projection}\;P_{ref}$, the feature point offset limit χ, and vote threshold λ.
**Output:** The refined set of feature points $T_{refined}$
1. $\mathrm{Establish}\;\mathrm{the}\;\mathrm{initial}\;\mathrm{feature}\;\mathrm{set}\;T_{main}$ and $T_{ref}$ by the SURF.
2. $\mathrm{Calculate}\;\mathrm{the}\;\mathrm{feature}\;\mathrm{angle}\;A(t_{main}^{i},t_{main}^{j})$$\;\mathrm{and}\;A(t_{ref}^{i},t_{ref}^{j})$ (1≤i≤N,1≤j≤N).
3. Calculate the upper and lower limits of feature angle based on (6) and (7) using the feature point offset limit χ.
4. $\mathrm{Evaluate}\;\mathrm{each}\;\mathrm{feature}\;\mathrm{point}\;t_{main}^{i}$$\;\mathrm{by}\;\mathrm{the}\;\mathrm{voting}\;\mathrm{function}\;G_{i}$ based on (5) and (8).
5. $\mathrm{Get}\;\mathrm{the}\;\mathrm{feature}\;\mathrm{points}\;\mathrm{after}\;\mathrm{rough}\;\mathrm{elimination}\;T_{rough}$ by the vote threshold λ based on (9).
6. Calculate the alignment vector D$\;\mathrm{based}\;\mathrm{on}\;(10)\;\mathrm{by}\;\mathrm{the}\;T_{rough}$.
7. $\mathrm{Move}\;\mathrm{the}\;\mathrm{main}\;\mathrm{projection}\;P_{main}$ by the alignment vector D.
8. Calculate the SSIM between the reference projection and the main projection moved in step 7 based on (11).
9. $\mathrm{Sort}\;\mathrm{the}\;\mathrm{feature}\;\mathrm{points}\;\mathrm{according}\;\mathrm{to}\;\mathrm{SSIM}.\;20\%\;\mathrm{points}\;\mathrm{of}\;T_{rough}$$\;\mathrm{are}\;\mathrm{built}\;\mathrm{as}\;\mathrm{the}\;\mathrm{refined}\;\mathrm{feature}\;\mathrm{set}\;T_{refined}$.

**Table 2:** The process of relocation based on SSIM.

**Input:**$\;\mathrm{The}\;\mathrm{refined}\;\mathrm{feature}\;\mathrm{set}\;T_{refined}$. Neighborhood image size γpixel×γpixel.
**Output:**$\mathrm{The}\;\mathrm{optimal}\;\mathrm{set}\;\mathrm{of}\;\mathrm{feature}\;\mathrm{points}\;T_{refined\_relocation}$.
1. $\mathrm{Crop}\;\mathrm{the}\;\mathrm{neighborhood}\;\mathrm{images}\;P_{main}^{\gamma }(x,y,t_{main}^{i})$$\;\mathrm{and}\;P_{ref}^{\gamma }(x,y,t_{ref}^{i})$ $\mathrm{with}\;\mathrm{the}\;\mathrm{center}\;\mathrm{of}\;t_{main}^{i}$$\;\mathrm{and}\;t_{ref}^{i}$ by the neighborhood image size γpixel×γpixel.
2. $\mathrm{Move}\;P_{main}^{\gamma }(x,y,t_{main}^{i})$$\;\mathrm{in}\;\mathrm{steps}\;\mathrm{of}\;0.1\;\mathrm{pixels}\;\mathrm{to}\;\mathrm{obtain}\;P_{main}^{\gamma }(x,y,t_{main}^{i})$.
3. $\mathrm{Calculate}\;\mathrm{the}\;\mathrm{SSIM}\;\mathrm{between}\;P_{main}^{\gamma }(x,y,t_{main}^{i,new})$$\;\mathrm{and}\;P_{ref}^{\gamma }(x,y)$.
4. $\mathrm{The}\;\mathrm{position}\;\mathrm{with}\;\mathrm{the}\;\mathrm{largest}\;\mathrm{SSIM}\;\mathrm{is}\;\mathrm{taken}\;\mathrm{as}\;\mathrm{the}\;\mathrm{new}\;\mathrm{feature}\;\mathrm{point}\;\mathrm{position}.\;\mathrm{Finally},\;\mathrm{the}\;\mathrm{feature}\;\mathrm{set}\;\mathrm{after}\;\mathrm{relocation}\;T_{refined\_relocation}$ is obtained.

**Table 3:** Scan sample and exposure time.

No.	Sample	Exposure Time (s)	Interval Time (s)
1	Wasp	10	4860
2	Cabbage seed	15	25,500
3	Bamboo stick	20	32,700
4	Star card	60	3660

**Table 4:** Scan parameters of 3D reconstruction experiment.

Parameter	Electronic Component	Cabbage Seed
Voltage (kV)	60	
Exposure time (s)	15	
Image size (pixel)	1065 × 1030	
Resolution (nm)	350	700
Main rotation step (°)	0.36	0.25
Reference rotation step (°)	3.6	2.5
Scanning time (h)	5.5	7.5

**Table 5:** Feature point elimination result.

Image Number	Sample	Method	Horizontal	Vertical	RMSE
1	Wasp	Ground truth	3.58	1.13	
		SURF	3.08	−1.33	1.78
		RANSAC	3.71	1.00	0.11
		LPM	3.85	0.99	0.21
		ASIM	3.63	1.14	0.038
2	Cabbage seed	Ground truth	0.89	2.77	
		SURF	0.41	1.09	1.24
		RANSAC	0.60	1.77	0.74
		LPM	0.84	2.26	0.36
		ASIM	0.91	2.88	0.079
3	Bamboo stick	Ground truth	20.75	11.99	
		SURF	11.67	8.96	6.77
		RANSAC	21.71	13.23	1.11
		LPM	22.11	11.68	0.99
		ASIM	20.60	12.35	0.27
4	Star card	Ground truth	41.93	90.14	
		SURF	59.64	63.61	22.56
		RANSAC	41.98	89.97	0.13
		LPM	44.67	83.98	4.77
		ASIM	41.91	90.18	0.034

## Data Availability

The data and the code used for the manuscript are available for researchers on request from the corresponding author.

## References

[1] Samber B.D., Renders J., Elberfeld T., Maris Y., Sijbers J. (2021). FleXCT: A Flexible X-ray CT scanner with 10 degrees of freedom. Opt. Express.

[2] Oliveira F.D., Maurício D., Nardelli V.C., Arenhart F.A., Donatelli G.D. (2014). Characterization and Correction of Geometric Errors Induced by Thermal Drift in CT Measurements. Key Eng. Mater..

[3] Vavřík D., Jandejsek I., Pichotka M. (2016). Correction of the X-ray tube spot movement as a tool for improvement of the micro-tomography quality. J. Instrum..

[4] Odstril M., Holler M., Raabe J., Guizar-Sicairos M. (2019). Alignment methods for nanotomography with deep subpixel accuracy. Opt. Express.

[5] Dierolf M., Menzel A., Thibault P., Schneider P., Kewish C.M., Wepf R., Bunk O., Pfeiffer F. (2010). Ptychographic X-ray computed tomography at the nanoscale. Nature.

[6] Guizar-Sicairos M., Diaz A., Holler M., Lucas M.S., Bunk O. (2011). Phase tomography from x-ray coherent diffractive imaging projections. Opt. Express.

[7] Kingston A., Sakellariou A., Varslot T., Myers G., Sheppard A. (2011). Reliable automatic alignment of tomographic projection data by passive auto-focus. Med Phys..

[8] Ramos T., Jrgensen J.S., Andreasen J.W. (2017). Automated angular and translational tomographic alignment and application to phase-contrast imaging. J. Opt. Soc. Am. A Opt. Image Sci. Vis..

[9] Huang X., Wild S.M., Di Z.W. Calibrating Sensing Drift in Tomographic Inversion. Proceedings of the 2019 IEEE International Conference on Image Processing (ICIP).

[10] Sasov A., Xuan L., Salmon P.L. (2008). Compensation of mechanical inaccuracies in micro-CT and nano-CT. Proc. SPIE-Int. Soc. Opt. Eng..

[11] Evangelidis G.D., Psarakis E.Z. (2008). Parametric Image Alignment Using Enhanced Correlation Coefficient Maximization. IEEE Trans. Pattern Anal. Mach. Intell..

[12] Manuel Guizar-Sicairos S.T.T., Fienup J.R. (2008). Efficient subpixel image registration algorithms. Opt. Lett..

[13] Lowe D.G. (2004). Distinctive Image Features from Scale-Invariant Keypoints. Int. J. Comput. Vis..

[14] Bay H., Ess A., Tuytelaars T., Gool L.V. (2008). Speeded-Up Robust Features (SURF). Comput. Vis. Image Underst..

[15] Calonder M. (2012). BRIEF: Computing a Local Binary Descriptor Very Fast. IEEE Trans. Pattern Anal. Mach. Intell..

[16] Ma J., Zhou H., Zhao J., Gao Y., Jiang J., Tian J. (2015). Robust Feature Matching for Remote Sensing Image Registration via Locally Linear Transforming. IEEE Trans. Geosci. Remote Sens..

[17] Ma J., Ji Z., Tian J., Yuille A.L., Tu Z. (2014). Robust Point Matching via Vector Field Consensus. IEEE Trans. Image Process..

[18] Pang S., Xue J., Qi T., Zheng N. (2014). Exploiting local linear geometric structure for identifying correct matches. Comput. Vis. Image Underst..

[19] Ma J., Ma Y., Zhao J., Tian J. (2014). Image Feature Matching via Progressive Vector Field Consensus. IEEE Signal. Process. Lett..

[20] Ma J., Zhao J., Jiang J., Zhou H., Guo X. (2019). Locality Preserving Matching. Int. J. Comput. Vis..

[21] Torr P., Zisserman A. (2000). MLESAC: A New Robust Estimator with Application to Estimating Image Geometry. Comput. Vis. Image Underst..

[22] Jian F., Chen L., Zhenzhong L., Roeder R.K. (2015). Analysis and Correction of Dynamic Geometric Misalignment for Nano-Scale Computed Tomography at BSRF. PLoS ONE.

[23] Flay N., Sun W., Brown S. (2015). Investigation of the Focal Spot Drift in Industrial Cone-Beam X-ray Computed Tomography. Digital Industrial Radiology and Computed Tomography (DIR). https://www.ndt.net/events/DIR2015/app/content/Paper/77_Sun.pdf.

[24] Zhou W., Bovik A.C., Sheikh H.R., Simoncelli E.P. (2004). Image quality assessment: From error visibility to structural similarity. IEEE Trans. Image Process..

[25] Hiller J., Maisl M., Reindl L.M. (2012). Physical characterization and performance evaluation of an X-ray micro-computed tomography system for dimensional metrology applications. Meas. Sci. Technol..

